# *trans*-Diamminedichloridobis(1*H*-imidazole-κ*N* ^3^)nickel(II)

**DOI:** 10.1107/S1600536813016747

**Published:** 2013-06-22

**Authors:** Piskala Subburaman Kannan, Ayyakannu Sundaram Ganeshraja, Kanniah Rajkumar, Krishnamoorthy Anbalagan, Arunachalatheva SubbiahPandi

**Affiliations:** a Department of Physics, S.M.K. Fomra Institute of Technology, Thaiyur, Chennai 603 103, India; b Department of Chemistry, Pondicherry University, Pondicherry 605 014, India; c Department of Physics, Presidency College (Autonomous), Chennai 600 005, India

## Abstract

The whole mol­ecule of the title compound, [NiCl_2_(C_3_H_4_N_2_)_2_(NH_3_)_2_], is generated by inversion symmetry. The Ni^II^ ion, which is located on an inversion center, has a distorted octa­hedral coordination environment and is surrounded by two ammine N atoms and two Cl atoms in the equatorial plane, with two N atoms of two imidazole groups occupying the axial positions. The imidazole ring makes a dihedral angle of 81.78 (18)° with the Ni/N/Cl equatorial plane. In the crystal, mol­ecules are linked *via* N—H⋯Cl hydrogen bonds and C—H⋯π inter­actions, forming a three-dimensional network.

## Related literature

For applications of imidazole and its derivatives, see: Huang *et al.* (2008 ▶, 2011 ▶). For the biological activity of imidazole deriv­atives, see: Gaonkar *et al.* (2009 ▶).
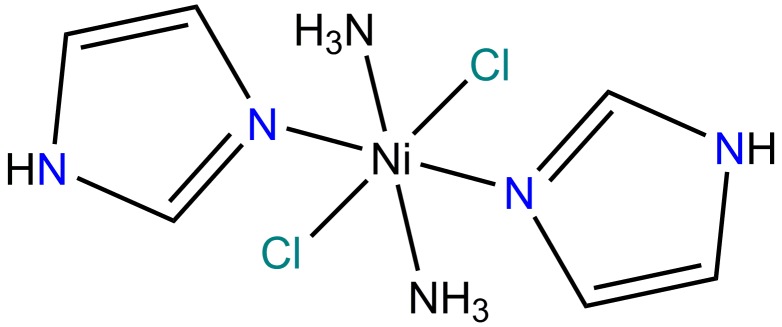


## Experimental

### 

#### Crystal data

[NiCl_2_(C_3_H_4_N_2_)_2_(NH_3_)_2_]
*M* *_r_* = 299.82
Orthorhombic, 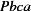

*a* = 9.1349 (9) Å
*b* = 7.9451 (5) Å
*c* = 15.6121 (13) Å
*V* = 1133.09 (16) Å^3^
*Z* = 4
Mo *K*α radiation
μ = 2.16 mm^−1^
*T* = 293 K
0.5 × 0.4 × 0.4 mm

#### Data collection

Oxford Diffraction Xcalibur Eos diffractometer
Absorption correction: multi-scan (*CrysAlis PRO*; Oxford Diffraction, 2009 ▶) *T*_min_ = 0.369, *T*_max_ = 0.421
4464 measured reflections
1338 independent reflections
1137 reflections with *I* > 2σ(*I*)
*R*_int_ = 0.017

#### Refinement

*R*[*F* ^2^ > 2σ(*F* ^2^)] = 0.045
*wR*(*F* ^2^) = 0.129
*S* = 1.11
1338 reflections
71 parameters
H-atom parameters constrained
Δρ_max_ = 0.76 e Å^−3^
Δρ_min_ = −1.00 e Å^−3^

### 

Data collection: *CrysAlis CCD* (Oxford Diffraction, 2009 ▶); cell refinement: *CrysAlis CCD*; data reduction: *CrysAlis RED* (Oxford Diffraction, 2009 ▶); program(s) used to solve structure: *SHELXS97* (Sheldrick, 2008 ▶); program(s) used to refine structure: *SHELXL97* (Sheldrick, 2008 ▶); molecular graphics: *ORTEP-3 for Windows* (Farrugia, 2012 ▶) and *PLATON* (Spek, 2009 ▶); software used to prepare material for publication: *SHELXL97* and *PLATON* (Spek, 2009 ▶).

## Supplementary Material

Crystal structure: contains datablock(s) global, I. DOI: 10.1107/S1600536813016747/su2612sup1.cif

Structure factors: contains datablock(s) I. DOI: 10.1107/S1600536813016747/su2612Isup2.hkl

Additional supplementary materials:  crystallographic information; 3D view; checkCIF report

## Figures and Tables

**Table 1:** Hydrogen-bond geometry (Å, °) *Cg*1 is the centroid of the N3/C4/N5/C6/C7 ring.

*D*—H⋯*A*	*D*—H	H⋯*A*	*D*⋯*A*	*D*—H⋯*A*
N5—H5⋯Cl2^i^	0.86	2.53	3.268 (3)	144
N8—H8*A*⋯Cl2^ii^	0.89	2.32	3.180 (3)	162
N8—H8*B*⋯Cl2^iii^	0.89	2.37	3.210 (3)	157
C4—H4⋯*Cg*1^iv^	0.93	2.95	3.772 (5)	148

Symmetry codes: (i) 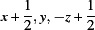; (ii) 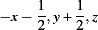; (iii) 
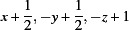
; (iv) 
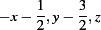
.

**Fig. 1.**
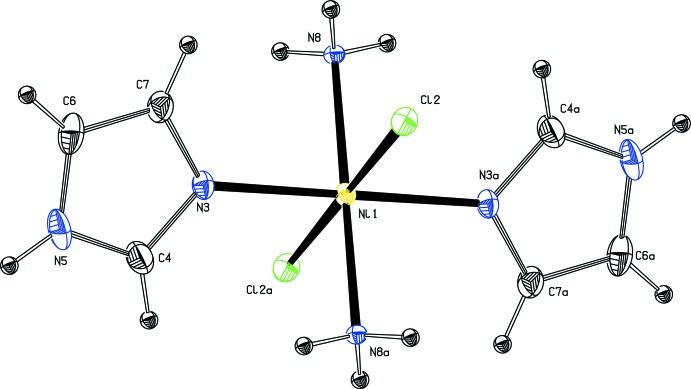
View of the molecular structure of the title compound, with atom labelling. Displacement ellipsoids are drawn at the 30% probability level.

**Fig. 2.**
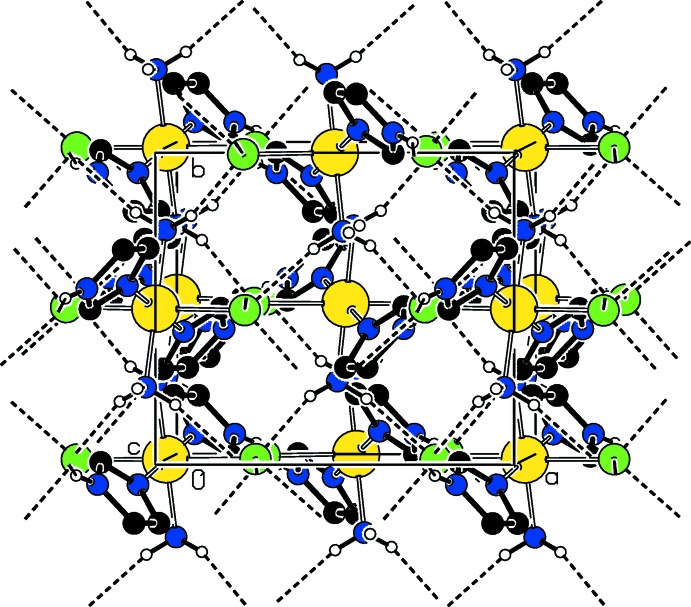
The crystal packing of the title compound viewed along the c axis. Dashed lines show the N—H···Cl hydrogen bonds [see Table 1 for details]

## supplementary crystallographic information

### Comment

Knowledge of the detailed coordination behaviour of imidazoles and their limitation in the possible use in complexes with specific catalytic activity is of great current importance. Because of their multiple coordination modes imidazole, namely 1,3-diazacyclopenta- 2,4-diene, and its derivatives have found a wide range of applications in coordination chemistry and for the construction of novel metal–organic frameworks (Huang *et al.*, 2008; Huang *et al.*, 2011).

The chemistry of imidazole occupies an extremely important position within the family of five-membered heterocyclic compounds. Synthesis of imidazole derivatives has attracted great interest in recent years due to their broad spectrum of biological activities (Gaonkar *et al.*, 2009). Herein we report on the crystal structure of the title compound.

The molecular structure of the title compound as illustrated in Fig. 1. The nickel(II) ion is located on an inversion center and has a distorted NiN4Cl2 octahedral coordination environment. It is surrounded by four N atoms, two of which are in the equatorial plane with the Cl atoms, and the remaining two N atoms occupy the axial positions. The imidazole ring (N3/N5/C4/C6/C7) is planar with a maximum deviation of -0.005 (1)Å for atom C4. It makes a dihedral angle of 81.78 (18) ° with the equatorial plane of atoms Ni/Cl2/N3/Cl2a/N3a [symmetry code: (a) -x, -y, -z+1].

In the crystal, molecules are linked via N-H···Cl hydrogen bonds and C-H···π interactions forming a three-dimensional network (Table 1 and Fig. 2).

### Experimental

A total of 10 mL of a 0.01 M aqueous solution of NiCl_2_ was slowly mixed with 20 mL of a 0.02 M ammonia solution. After 1 h, 20 mL of a 0.02 M aqueous solution of imidazole was added drop wise. The mixture was slowly evaporated at room temperature, and deep-green block-like crystals of the title complex were obtained within 5 days. The crystals were filtered, washed with water, and dried in a desiccator over P_4_O_10_.

### Refinement

All the H atoms were fixed geometrically and allowed to ride on their parent N or C atoms: N-H = 0.86 and 0.89 Å for NH and NH_3_ H atoms, respectively, C—H = 0.93–0.97 Å; U_iso_(H) = 1.5U_eq_(C-methyl) and = 1.2U_eq_(N,C) for other H atoms.

### Crystal data

**Table:**

[NiCl_2_(C_3_H_4_N_2_)_2_(NH_3_)_2_]	*F*(000) = 616
*M**_r_* = 299.82	*D*_x_ = 1.758 Mg m^−^^3^
Orthorhombic, *P**b**c**a*	Mo *K*α radiation, λ = 0.71073 Å
Hall symbol: -P 2ac 2ab	Cell parameters from 1338 reflections
*a* = 9.1349 (9) Å	θ = 5.2–29.1°
*b* = 7.9451 (5) Å	µ = 2.16 mm^−^^1^
*c* = 15.6121 (13) Å	*T* = 293 K
*V* = 1133.09 (16) Å^3^	Block, green
*Z* = 4	0.5 × 0.4 × 0.4 mm

### Data collection

**Table:**

Oxford Diffraction Xcalibur Eos diffractometer	1338 independent reflections
Radiation source: fine-focus sealed tube	1137 reflections with *I* > 2σ(*I*)
Graphite monochromator	*R*_int_ = 0.017
Detector resolution: 15.9821 pixels mm^-1^	θ_max_ = 29.1°, θ_min_ = 5.2°
ω and φ scan	*h* = −8→12
Absorption correction: multi-scan (*CrysAlis PRO*; Oxford Diffraction, 2009)	*k* = −10→10
*T*_min_ = 0.369, *T*_max_ = 0.421	*l* = −21→15
4464 measured reflections	

### Refinement

**Table:**

Refinement on *F*^2^	Primary atom site location: structure-invariant direct methods
Least-squares matrix: full	Secondary atom site location: difference Fourier map
*R*[*F*^2^ > 2σ(*F*^2^)] = 0.045	Hydrogen site location: inferred from neighbouring sites
*wR*(*F*^2^) = 0.129	H-atom parameters constrained
*S* = 1.11	*w* = 1/[σ^2^(*F*_o_^2^) + (0.0565*P*)^2^ + 3.9561*P*] where *P* = (*F*_o_^2^ + 2*F*_c_^2^)/3
1338 reflections	(Δ/σ)_max_ < 0.001
71 parameters	Δρ_max_ = 0.76 e Å^−^^3^
0 restraints	Δρ_min_ = −1.00 e Å^−^^3^

### Special details

**Table:**

Geometry. All esds (except the esd in the dihedral angle between two l.s. planes) are estimated using the full covariance matrix. The cell esds are taken into account individually in the estimation of esds in distances, angles and torsion angles; correlations between esds in cell parameters are only used when they are defined by crystal symmetry. An approximate (isotropic) treatment of cell esds is used for estimating esds involving l.s. planes.
Refinement. Refinement of F^2^ against ALL reflections. The weighted R-factor wR and goodness of fit S are based on F^2^, conventional R-factors R are based on F, with F set to zero for negative F^2^. The threshold expression of F^2^ > 2sigma(F^2^) is used only for calculating R-factors(gt) etc. and is not relevant to the choice of reflections for refinement. R-factors based on F^2^ are statistically about twice as large as those based on F, and R- factors based on ALL data will be even larger.

### Fractional atomic coordinates and isotropic or equivalent isotropic displacement parameters (Å^2^)

**Table:**

	*x*	*y*	*z*	*U*_iso_*/*U*_eq_	
C4	0.1725 (5)	0.0044 (5)	0.3343 (3)	0.0331 (9)	
H4	0.2329	−0.0824	0.3530	0.040*	
C6	0.0749 (5)	0.1968 (6)	0.2518 (3)	0.0369 (9)	
H6	0.0539	0.2659	0.2053	0.044*	
C7	0.0103 (4)	0.1991 (5)	0.3298 (2)	0.0288 (8)	
H7	−0.0643	0.2720	0.3462	0.035*	
Cl2	−0.25211 (9)	−0.00290 (10)	0.44598 (5)	0.0227 (2)	
N3	0.0715 (3)	0.0774 (4)	0.38102 (17)	0.0209 (6)	
N5	0.1766 (4)	0.0725 (5)	0.2555 (2)	0.0372 (8)	
H5	0.2338	0.0425	0.2145	0.045*	
N8	−0.0253 (3)	0.2503 (3)	0.53954 (16)	0.0128 (5)	
H8A	−0.0989	0.2973	0.5109	0.015*	
H8B	0.0568	0.3070	0.5292	0.015*	
H8C	−0.0446	0.2530	0.5954	0.015*	
Ni1	0.0000	0.0000	0.5000	0.0150 (2)	

### Atomic displacement parameters (Å^2^)

**Table:**

	*U*^11^	*U*^22^	*U*^33^	*U*^12^	*U*^13^	*U*^23^
C4	0.031 (2)	0.041 (2)	0.0275 (19)	0.0049 (16)	0.0083 (16)	0.0018 (15)
C6	0.040 (2)	0.050 (2)	0.0203 (16)	−0.0078 (19)	0.0006 (16)	0.0116 (18)
C7	0.0277 (19)	0.035 (2)	0.0233 (17)	0.0025 (15)	0.0011 (14)	0.0094 (15)
Cl2	0.0183 (4)	0.0255 (4)	0.0242 (4)	−0.0017 (3)	−0.0041 (3)	0.0038 (3)
N3	0.0209 (14)	0.0267 (14)	0.0152 (12)	−0.0010 (11)	0.0022 (11)	0.0021 (11)
N5	0.0391 (19)	0.052 (2)	0.0206 (14)	−0.0066 (17)	0.0140 (14)	−0.0034 (15)
N8	0.0144 (11)	0.0118 (10)	0.0123 (10)	0.0009 (9)	−0.0010 (9)	−0.0004 (9)
Ni1	0.0151 (3)	0.0177 (3)	0.0123 (3)	0.00026 (19)	−0.00016 (19)	0.00066 (19)

### Geometric parameters (Å, º)

**Table:**

C4—N3	1.312 (5)	N3—Ni1	2.063 (3)
C4—N5	1.345 (5)	N5—H5	0.8600
C4—H4	0.9300	N8—Ni1	2.095 (2)
C6—C7	1.353 (6)	N8—H8A	0.8900
C6—N5	1.357 (6)	N8—H8B	0.8900
C6—H6	0.9300	N8—H8C	0.8900
C7—N3	1.374 (5)	Ni1—N3^i^	2.063 (3)
C7—H7	0.9300	Ni1—N8^i^	2.095 (2)
Cl2—Ni1	2.4527 (9)	Ni1—Cl2^i^	2.4527 (9)
			
N3—C4—N5	110.5 (4)	Ni1—N8—H8C	109.5
N3—C4—H4	124.7	H8A—N8—H8C	109.5
N5—C4—H4	124.7	H8B—N8—H8C	109.5
C7—C6—N5	105.7 (3)	N3^i^—Ni1—N3	180.0
C7—C6—H6	127.1	N3^i^—Ni1—N8	89.00 (11)
N5—C6—H6	127.1	N3—Ni1—N8	91.00 (11)
C6—C7—N3	109.7 (4)	N3^i^—Ni1—N8^i^	91.00 (11)
C6—C7—H7	125.2	N3—Ni1—N8^i^	89.00 (11)
N3—C7—H7	125.2	N8—Ni1—N8^i^	180.0
C4—N3—C7	105.9 (3)	N3^i^—Ni1—Cl2^i^	89.45 (8)
C4—N3—Ni1	126.3 (3)	N3—Ni1—Cl2^i^	90.55 (8)
C7—N3—Ni1	127.2 (2)	N8—Ni1—Cl2^i^	89.62 (7)
C4—N5—C6	108.2 (3)	N8^i^—Ni1—Cl2^i^	90.38 (7)
C4—N5—H5	125.9	N3^i^—Ni1—Cl2	90.55 (8)
C6—N5—H5	125.9	N3—Ni1—Cl2	89.45 (8)
Ni1—N8—H8A	109.5	N8—Ni1—Cl2	90.38 (7)
Ni1—N8—H8B	109.5	N8^i^—Ni1—Cl2	89.62 (7)
H8A—N8—H8B	109.5	Cl2^i^—Ni1—Cl2	180.0
			
N5—C6—C7—N3	−0.1 (5)	C4—N3—Ni1—N8	143.9 (3)
N5—C4—N3—C7	−0.9 (5)	C7—N3—Ni1—N8	−46.1 (3)
N5—C4—N3—Ni1	170.8 (3)	C4—N3—Ni1—N8^i^	−36.1 (3)
C6—C7—N3—C4	0.6 (5)	C7—N3—Ni1—N8^i^	133.9 (3)
C6—C7—N3—Ni1	−171.1 (3)	C4—N3—Ni1—Cl2^i^	54.3 (3)
N3—C4—N5—C6	0.9 (5)	C7—N3—Ni1—Cl2^i^	−135.7 (3)
C7—C6—N5—C4	−0.5 (5)	C4—N3—Ni1—Cl2	−125.7 (3)
C4—N3—Ni1—N3^i^	126 (8)	C7—N3—Ni1—Cl2	44.3 (3)
C7—N3—Ni1—N3^i^	−64 (8)		

Symmetry code: (i) −*x*, −*y*, −*z*+1.

### Hydrogen-bond geometry (Å, º)

Cg1 is the centroid of the N3/C4/N5/C6/C7 ring.

**Table:**

*D*—H···*A*	*D*—H	H···*A*	*D*···*A*	*D*—H···*A*
N5—H5···Cl2^ii^	0.86	2.53	3.268 (3)	144
N8—H8*A*···Cl2^iii^	0.89	2.32	3.180 (3)	162
N8—H8*B*···Cl2^iv^	0.89	2.37	3.210 (3)	157
C4—H4···*Cg*1^v^	0.93	2.95	3.772 (5)	148

Symmetry codes: (ii) *x*+1/2, *y*, −*z*+1/2; (iii) −*x*−1/2, *y*+1/2, *z*; (iv) *x*+1/2, −*y*+1/2, −*z*+1; (v) −*x*−1/2, *y*−3/2, *z*.
